# Possibility of live birth in patients with low serum β-hCG 14 days after blastocyst transfer

**DOI:** 10.1186/s13048-020-00732-6

**Published:** 2020-11-12

**Authors:** Yixuan Wu, Haiying Liu

**Affiliations:** 1 Department of Obstetrics and Gynecology, Center for Reproductive Medicine/Department of Fetal Medicine and Prenatal Diagnosis/BioResource Research Center, Key Laboratory for Major Obstetric Diseases of Guangdong Province, The Third Affiliated Hospital of Guangzhou Medical University, Guangzhou, China; 2 Key Laboratory of Reproductive Medicine of Guangdong Province, No. 63, Duobao Road, Guangzhou, Guangdong China; 3 Key Laboratory for Major Obstetric Diseases of Guangdong Province, No. 63, Duobao Road, Guangzhou, Guangdong China; 4 Key Laboratory of Reproduction and Genetics of Guangdong Higher Education Institutes, No. 63, Duobao Road, Guangzhou, Guangdong China

**Keywords:** Assisted reproductive technology, Human chorionic gonadotropin, Pregnancy, Live birth, Blastocyst

## Abstract

**Background:**

Although prior work has attempted to predict pregnancy outcomes by assaying serum β-hCG levels after blastocyst transfer, no study has focused on pregnancy outcomes in those with initially low serum β-hCG levels. This study sought to investigate pregnancy outcomes of patients with low serum β-hCG levels 14 days after blastocyst transfer.

**Methods:**

A retrospective study was conducted at the Third Affiliated Hospital of Guangzhou Medical University to study patients whose serum β-hCG levels were at 5–299 mIU/ml 14 days after frozen blastocyst transfer. Rates of live birth, early miscarriage, biochemical pregnancy loss and ectopic pregnancy were analyzed according to the female patients’ age by Chi-squared analysis. Receiver operating characteristic (ROC) curves were plotted to explore the threshold of predicting clinical pregnancy and live births.

**Results:**

312 patients had serum β-hCG levels < 300 mIU/ml at 14 days after frozen blastocyst transfer, among which, 18.6% were live births, 47.4% were early miscarriages, 22.8% were biochemical pregnancies and 9.6% were ectopic pregnancies. ROC curve analysis showed that a predicted value of β-hCG for clinical pregnancy was 58.8 mIU/ml with an area under the ROC curve (AUC) of 0.752, a sensitivity of 95.0% and specificity of 53.5%. The threshold for live births was 108.6 mIU/ml with an AUC of 0.649, a sensitivity of 93.1% and a specificity of 37.0%. For the β-hCG fold increase over 48 h, the cut-off for clinical pregnancy was 1.4 with an AUC of 0.899, a sensitivity of 90.3% and a specificity of 77.8%. The threshold for live birth was 1.9 with an AUC of 0.808, a sensitivity of 88.5% and specificity of 64.5%.

**Conclusions:**

Initially low serum β-hCG levels 14 days after frozen blastocyst transfer indicated minimal chances of live birth. For patients having an initial β-hCG > 58.8 mIU/ml, luteal phase support should continue. Another serum β-hCG test and ultrasound should be performed one week later. When an initial serum β-hCG is < 58.8 mIU/ml, luteal phase support should be discontinued and serum β-hCG measured with ultrasound one week later.

## Introduction

Human chorionic gonadotropin (hCG) is secreted by syncytiotrophoblasts at the time of implantation. Since it is detected in maternal serum as soon as 6–8 days after fertilization, β-hCG is widely used in the clinic as a marker of pregnancy. In normal conception, β-hCG levels are doubled every 48 h, and consequently, this increased pattern is applied to discriminate normal pregnancy from a state of pathological pregnancy [1].

It is routine to have serum β-hCG tests conducted 9–14 days after embryo transfer to confirm the diagnosis of pregnancy. Many prior studies have explored the relationship between serum β-hCG levels and pregnancy outcomes. In fresh embryo transfer cycles, the thresholds of serum β-hCG levels to predict clinical pregnancy and live births were 111–213 IU/L and 160–222.8 IU/L respectively 10–12 days after transfer [2–7]. For frozen embryo transfer, the cut-off value was 137–399 IU/L for clinical pregnancy and 212–411 IU/L for live births 11–12 days after embryo transfer [6–8]. Higher β-hCG levels are indicative of better pregnancy outcomes including higher rates of clinical pregnancy and live births [9].

Although many previous studies have investigated the prediction of pregnancy outcomes by measuring serum β-hCG levels after blastocyst transfer, no study has yet focused on pregnancy outcomes of patients with an initially low serum β-hCG level. These patients are often in a state of anxiety, and tend to be concerned about the possibility of a poor prognosis. In order to follow pregnancy outcomes, an increased number of serum β-hCG tests are required; however, no appropriate follow-up plans have been suggested according to current scientific research.

This study sought to investigate pregnancy outcomes of patients with an initially low serum β-hCG level at 14 days after frozen blastocyst transfer. Prediction of pregnancy outcomes is performed with the intent of developing an appropriate clinical follow-up strategy.

## Materials and methods

### Population

This retrospective study included patients that had received a frozen blastocyst transfer in the Department of Reproductive Medicine of the Third Affiliated Hospital, Guangzhou Medical University (Guangzhou, China), between January 2014 and October 2019. Low serum hCG levels were defined as the lowest 5 percentile (i.e., 5–299 mIU/ml) of serum hCG levels in all pregnant women after frozen blastocyst transfer during the same period. Totally 8788 patients with serum β-hCG >25mIU/mL 14 days after frozen blastocyst transfer were screened and 312(3.5%) of them with low serum β-hCG levels (i.e., 5–299 mIU/ml) were included in the study. The study was approved by the local Ethics Committee of the Third Affiliated Hospital of Guangzhou Medical University.

### Assisted reproductive technology (ART) techniques and treatment protocols

Vitrification and thawing kits (Kitazato Biophama Co. Ltd. Shizuoka, Japan) were applied for blastocyst cryopreservation and thawing. For vitrification, the blastocysts were equilibrated in an Equilibration Solution for 2 min and then transferred to a Vitrification Solution, wherein the embryos would remain for 45–60s at 37 °C. Then the blastocysts were placed into a Cryotop and placed immediately into liquid nitrogen. For thawing blastocysts, the top of the Cryotop containing the embryos was placed in a Thawing Solution for 1 min at 37 °C. Next, embryos were transferred sequentially to a Diluent Solution, a Washing Solution 1, and then Washing Solution 2 where they respectively remained for 3 min per sequential step at room temperature.

### Endometrial preparation and embryo transfer

Three protocols were available for endometrial preparation: 1) the natural cycle, 2) artificial cycle and 3) ovarian stimulation cycle. For patients with a regular menstruation cycle, the natural cycle was the first choice and blastocysts were transferred 5 days post-ovulation. For patients without follicular development, an artificial cycle was given. Oral estrogen (Estradiol Valerate, Bayer, Germany) at 3 mg given twice daily, was started at 2–4 days of the cycle and continued for at least 7 days. When an endometrial thickness was ≥7 mm, vaginal progesterone (Crinone, Merck Serono, Germany) was given at 90 mg once a day for 5 days; following which, blastocysts were transferred on day 6. For the stimulation cycle, 37.5–75 IU of human menopausal gonadotropin (HMG) was administered for 2–4 days of the cycle. When the dominant follicle was ≥18 mm, 8000–10,000 IU of human chorionic gonadotropin (HCG) was given to induce ovulation. Blastocyst transfer was done 5 days post-ovulation. Vaginal progesterone (Crinone, Merck Serono, Germany 90 mg qd) was given for luteal phase support in artificial cycles. For natural and stimulatory cycles, vaginal progesterone gels (Utrogestan, Besins Healthcare, France, 200 mg bid) was used to support the luteal phase. Serum β-hCG assays were done 14 days after embryo transfer. Luteal phase support was continued to week 10 when β-hCG tests were positive.

### Hormone measurement

An immunochemiluminometric assay was undertaken to assay for β-hCG (Architech i2000SR; Abbott Laboratories Inc., Chicago, IL, USA). The detection range lied between 1.2 and 225,000 mIU/ml. The sensitivity of the assay was 1.2 mIU/ml, with an intra-assay coefficient of variation of 7%. Our laboratory is annually screened for qualification by the External Quality Assessment of a Clinical Laboratory Center (Ministry of Health of the People’s Republic of China, Beijing, China). Fold increases in β-hCG concentrations over 48 h were calculated by the following formula: Fold increase = $$ {\left(\frac{\mathrm{HCG}1}{\mathrm{HCG}0}\right)}^{\raisebox{1ex}{$2$}\!\left/ \!\raisebox{-1ex}{$\mathrm{days}$}\right.} $$. HCG0 was defined as serum β-hCG concentrations that were measured 14 days after embryo transfer; HCG1 was defined as serum β-hCG levels in the second test, and the term “days” represented the interval between the two β-hCG tests.

### Definitions of pregnancy outcomes

Clinical pregnancy was defined as an intrauterine/extrauterine gestational sac that was detected by ultrasound with positive serum β-hCG levels. Biochemical pregnancy loss was defined as a serum β-HCG level > 5mIU/ml 14 days after transferring the embryo, which declined to < 5 mIU/ml at the end of pregnancy without a visible gestational sac by ultrasound. Early miscarriage was defined as fetal growth arrest or no cardiac activity that was detected in the gestational sac during the first 12 weeks of pregnancy. Live birth indicated a pregnancy that continued after 28 weeks of gestation with a live fetus evident.

### Statistics

Statistical analysis was performed with SPSS version 22.0 software (IBM, Armonk, NY, USA). Quantitative variables with a homogenous variance were expressed as ^−^X ± SD and the means were compared by the Student’s t-test. Quantitative variables with a heterogeneous variance were expressed as the median (1st and 3rd quartiles), and the medians were compared by the Mann-Whitney U test. A Chi-squared test was used to compare rates. Fisher’s exact test was applied when the expected count was < 5 or the total sample size was < 40. The effect of serum β-hCG on pregnancy outcomes was explored by logistic regression analysis. Serum β-hCG levels and fold changes in β-hCG levels over 48 h were applied to predict clinical pregnancy as well as live births by plotting Receiver Operating Characteristic (ROC) curves. An alpha value of *P* < 0.05 was considered statistically significant.

## Results

### Pregnancy outcomes of patients with low serum β-hCG

A total of 312 patients had serum β-hCG levels < 300 mIU/ml 14 days after blastocyst transfer, among which, 18.6% were live births, 47.4% were early miscarriages, 22.8% were biochemical pregnancies and 9.6% were ectopic pregnancies. Rates of biochemical pregnancy loss, ectopic pregnancy, early and late miscarriages, live birth were comparable between the < 38 years group, and the > 38 years group (Table 1). Among the 241 clinical pregnancies, 225 (93.4%) were singletons and 16 (6.6%) were twins (i.e., 9 monozygotic twins and 7 dizygotic twins). The rate of live birth was 24.9% (56/225) in singletons and 12.5% (2/16) in twins. The lower limits of serum-borne β-hCG levels were 64.9 mIU/ml for singleton live births, 145.1 mIU/ml for twin live births, 15.3 mIU/ml for early miscarriage, and 5.3 mIU/ml for ectopic pregnancies.

**Table 1:** Pregnancy outcomes of patients with serum β-hCG level < 300 mIU/ml 14 days after blastocyst transfer

Pregnancy outcomes	< 38 years	≥38 years	Total	*P*
	%(n)	%(n)	%(n)	
Female age (years)	31 (28,34)	39 (38,41)		1.13E-46 ^a^
AMH (ng/ml)	5.83 (3.56, 9.34)	3.64 (1.84,6.00)		7.45E-5
BMI (kg/m^2^)	21.9 ± 3.2	23.6 ± 3.7		0.001
Days of embryos transfer %(n)				0.645
5	68.2 (176/258)	55.6 (30/54)		
6	29.5 (76/258)	44.4 (24/54)		
both 5&6	2.3 (6/258)	0(0/54)		
No. of embryos transferred % (n)				0.954
1	43.0 (111/258)	42.6 (23/54)		
2	57.0 (147/258)	57.4 (31/54)		
Biochemical pregnancy loss	22.5 (58)	24.1 (13)	22.8 (71)	0.800
Live birth % (n)	19.0 (49)	16.6 (9)	18.6 (58)	0.690
Ectopic pregnancy % (n)	10.1 (26)	7.4 (4)	9.6 (30)	0.545
Early Miscarriage % (n)	46.9 (121)	50.0 (27)	47.4 (148)	0.678
Late miscarriage % (n)	1.5 (4)	1.9 (1)	1.6 (5)	1.000 ^b^

a *P* values meant to compare differences between the groups of < 38 years and ≥ 38 years .b Fisher exact test were used

In sum, 164 patients had another serum β-hCG test at 2–24 days (mean: 6.75 days) after the initial measurement, among which, 133 had increased β-hCG levels and 31 had decreased values. For patients with decreased β-hCG levels, 96.8% (30/31) were biochemical pregnancy loss. The only patient, although presenting with declined β-hCG levels from 133.4 to 64.5 mIU/ml, eventually developed into an ectopic pregnancy.

### Pregnancy outcomes of patients with different β-hCG intervals

For patients with β-hCG levels of 5–50 mIU/ml, no live birth occurred. Most of them were biochemical pregnancies (77.8%) and the remainder were early miscarriages (13.9%) and ectopic pregnancies (8.3%). Among patients with β-hCG levels of 51–100 mIU/ml, it was found that 55.8% were early miscarriages, 25.0% were biochemical pregnancies, and only 7.7% were live births. For patients with β-hCG levels of 101–200 and 201–299 mIU/ml, the likelihood of live births was about 1/4 (i.e., 23.7 and 24.5%, respectively) and the probability of early miscarriage was about 1/2 (i.e., 50 and 51.9% respectively; Table 2).

**Table 2:** Pregnancy outcomes of patients with different β-hCG levels 14 days after blastocyst transfer

HCG level mIU/ml	5–50	51–100	101–200	201–299	***P***
Female age (years)	33.0 ± 4.5	32.2 ± 4.2	32.2 ± 4.8	32.6 ± 5.2	0.835
AMH (ng/ml)	6.18 ± 4.37	6.79 ± 4.30	6.80 ± 4.78	6.60 ± 4.89	0.922
BMI (kg/m^2^)	21.6 ± 2.9	22.5 ± 3.0	21.9 ± 3.2	22.6 ± 3.8	0.269
Days of embryos transfer %(n)					0.356
5	63.6 (21/33)	75.9 (41/54)	58.8 (70/119)	69.8 (74/106)	
6	33.3 (11/33)	24.1 (13/54)	38.7 (46/119)	28.3 (30/106)	
both 5&6	3.0 (1/33)	0 (0/54)	2.5 (3/119)	1.9 (2/106)	
No. of embryos transferred % (n)					0.609
1	45.5 (15/33)	44.4 (24/54)	46.2 (55/119)	37.7 (40/106)	
2	54.5 (18/33)	55.6 (30/54)	53.8 (64/119)	62.3 (66/106)	
Serum β-hCG (mIU/ml)	34 (29,41)	80 (61,90)	145 (127,174)	244 (218,244)	1.93E-149
Biochemical pregnancy loss %(n)	75.8 (25/33)	29.6 (16/54)	13.4 (16/119)	13.2 (14/106)	3.81E-14
Early miscarriage %(n)	15.2 (5/33)	53.7 (29/54)	49.6 (59/119)	51.9 (55/106)	0.001
Ectopic pregnancy %(n)	9.0 (3/33)	7.4 (4/54)	11.8 (14/119)	8.5 (9/106)	0.827^a^
Late miscarriage %(n)	0.0 (0/33)	1.9 (1/54)	1.7 (2/119)	1.9 (2/106)	1.000^a^
Live birth %(n)	0.0 (0/33)	7.4 (4/54)	23.5 (28/119)	24.5 (26/106)	0.001

a Fisher exact test were used

### Characteristics of live birth vs. non-live birth

Baseline characteristics, including female and male age, number of prior pregnancies and previous transfers, anti-müllerian hormone (AMH), and body massive index (BMI) were comparable for both groups. There were no statistical differences between the groups in terms of the number of embryos transferred, the protocols of endometrial preparation, days of embryo transfer and endometrial thickness. However, the serum β-hCG levels of patients with live births (median: 196 mIU/ml) was significantly higher than that of patients with non-live births (median: 140 mIU/ml, *P* = 0.0001; Table 3).

**Table 3:** Characteristics of live birth vs. non-live birth

Characteristics	Live birth	Non-live birth	***P***
N	58	254	
Female age (years)	31.9 ± 4.7	32.5 ± 4.9	0.346
Male age (years)	34.8 ± 5.1	34.7 ± 5.1	0.837
Infertility duration (years)	4.6 ± 2.7	4.8 ± 3.5	0.625
No. of previous gestation			0.242
%(n)			
0	48.3 (28)	42.2 (107)	
1–2	48.3 (28)	47.6 (121)	
≥ 3	3.4 (2)	10.2 (26)	
Causes of Infertility %(n)			0.805
Tubal/ Peritoneal	55.2 (32)	48.0 (122)	
Ovulatory dysfunction	8.6 (5)	10.2 (26)	
Male factor	10.3 (6)	12.6 (32)	
Others	25.9 (15)	29.1 (74)	
No. of previous transfer	1.8 ± 1.0	2.1 ± 1.3	0.187
AMH (ng/ml)	6.80 ± 5.29	6.63 ± 4.54	0.807
BMI (kg/m^2^)	21.9 ± 2.9	22.3 ± 3.5	0.434
Types of cycle			0.378
Natural cycle	22.4 (13)	27.6 (70)	
Artificial cycle	77.6 (45)	70.5 (179)	
Stimulation cycle	0.0 (0)	2.0 (5)	
EMT^a^ 5 days before transfer (mm)	8.6 (8.0,10.0)	8.5 (7.6,10.0)	0.327
Days of embryos transfer %(n)			0.807
5	58.6 (34)	67.7 (172)	
6	36.2 (21)	31.1 (79)	
both 5&6	5.2 (3)	1.2 (3)	
No. of embryos transferred % (n)			0.574
1	39.7 (23)	43.7 (111)	
2	60.3 (35)	56.3 (143)	
Serum P^**b**^	65.8 ± 29.4	53.1 ± 28.7	0.115
Serum E_2_^**b**^	1125 (479,1386)	891 (472,1871)	0.820
Serum β-hCG (mIU/ml)	196 (144, 221)	140 (84,216)	2.40E-5

a *EMT* endometrium thickness. b Only 68 patients were tested for serum progesterone levels and 62 were tested for serum estradiol

### Effect of serum β-hCG levels on pregnancy outcomes

Serum β-hCG levels had a positive effect on pregnancy outcomes (OR for a 50 mIU/ml interval), including clinical pregnancy (OR 1.875; 95% CI 1.522–2.310; *P* = 0.0001), and live births (OR 1.416; 95% CI 1.162–1.726; *P* = 0.001; Table 4).

**Table 4:** Effects of serum β-hCG levels on pregnancy outcomes by logistic regression analysis

		95% Confidence Interval for OR		
	OR	lower limit	upper limit	*P*
Clinical pregnancy	1.875	1.522	2.310	1.38E-10
Early miscarriage	0.889	0 .739	1.069	0.889
Ectopic pregnancy	1.019	0.802	1.295	0.876
Live birth	1.416	1.162	1.726	0.001

Serum β-hCG was included as a categorical variable (categorized by 5–50, 51–100, 101–150, 151–200, 201–250, 251–299 mIU/ml)

Adjusted for female age, days of embryo transfer and number of embryo transfer

### Prediction of pregnancy outcomes

**Fig. 1:**
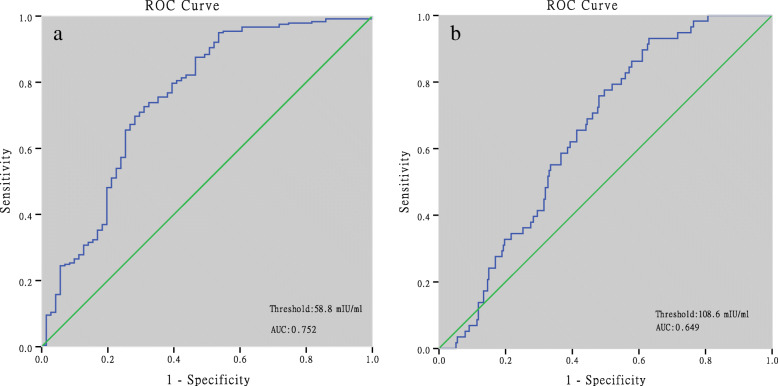
ROC curves for predicting pregnancy outcomes by serum β-hCG levels 14 days after blastocyst transfer. a: ROC curve analysis of β-hCG for clinical pregnancies; b: ROC curve analysis of β-hCG for live births

**Fig. 2:**
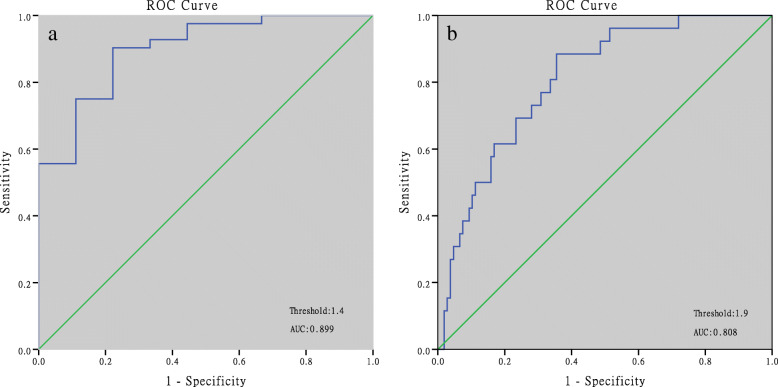
ROC curves for predicting pregnancy outcomes by fold increases in serum β-hCG levels over 48 h. a: ROC curve of the β-hCG fold increase for clinical pregnancies; b: ROC curve analysis of a β-hCG fold increase for live births

ROC analysis showed that the predicted value for clinical pregnancy was 58.8 mIU/ml with an AUC of 0.752 (95% CI: 0.680–0.823), a sensitivity of 95.0%, and a specificity of 53.5%. The threshold for live births was 108.6 mIU/ml with an AUC of 0.649 (95% CI: 0.0.583–0.715), a sensitivity of 93.1%, and a specificity of 37.0% (Fig. 1, Table 5). For the β-hCG fold increase over 48 h, the cut-off for a clinical pregnancy was 1.4 with an AUC of 0.899 (95% CI: 0.801–0.996), a sensitivity of 90.3%, and a specificity of 77.8%. The threshold for live births was 1.9 with an AUC of 0.808 (95% CI: 0.724–0.891), a sensitivity of 88.5%, and a specificity of 64.5% (Fig. 2, Table 5).

**Table 5:** Thresholds of serum β-hCG level for prediction of clinical pregnancies and live births

Pregnancy outcomes	Clinical pregnancy	Live birth	Clinical pregnancy	Live birth
	Threshold of β-hCG level (mIU/ml)		Threshold of β-hCG fold increase over 48 h	
	58.8	108.6	1.4	1.9
AUC	0.752	0.649	0.899	0.808
95% CI of AUC	0.680–0.823	0.583–0.715	0.801–0.996	0.724–0.891
Sensitivity %	95.0	93.1	90.3	88.5
Specificity %	53.5	37.0	77.8	64.5
PPV^a^ %	85.8	25.2	97.4	63.6
NPV^b^ %	73.3	95.9	33.3	92.3

a *PPV* positive predicted value; b *NPV* negative predicted value

## Discussion

In the present study, pregnancy outcomes of patients whose serum β-hCG levels were < 300 mIU/ml 14 days after blastocyst transfer were investigated. Our study showed that pregnancy outcomes of the patients with initially low serum β-hCG levels were poor, with only 18.6% of live births. Nearly 50% (47.4%) of the patients were early miscarriage and the rate of ectopic pregnancy was as high as 9.6%. Our research had merely included patients with initially low serum β-hCG levels, instead of all pregnant women, since there have been many studies like that. Patients with low β-hCG values are often very anxious about their pregnancy outcomes. In addition, they are often required to take serum β-hCG tests in order to monitor the progress of conception, which will increase the number of visits and thus bring them both psychological and economic stress.

For these reasons, it is very important to make individualized follow-up plans according to different serum β-hCG intervals. For patients with an initially low β-hCG, the most important consideration is to determine whether it is a clinical pregnancy. Our study demonstrated that the initial β-hCG value > 58.8 mIU/ml predicted 85.8% of clinical pregnancies, while a failure to achieve that value led to 73.3% of biochemical pregnancy loss. Among patients having a clinical pregnancy, about 60% were early miscarriage and 12.4% were ectopic pregnancy (data not shown). Therefore, for patients having an initial β-hCG > 58.8 mIU/ml, although luteal phase support is suggested to continue, an additional serum β-hCG test and ultrasound should be performed one week later to rule out an ectopic pregnancy. If the initial serum β-hCG is < 58.8 mIU/ml, it is suggested that luteal phase support is discontinued and measurement of serum β-hCG and ultrasound can be arranged one week later, since no live births occurred in this group of patients.

In our research, we calculated the β-hCG fold increase over 48 h according to the second test and found that a value of 1.9 was the optimal threshold to discriminate live births from non-live births. The probability of live birth was 63.6% if the fold increase was > 1.9, as compared with the minimal likelihood of live birth (7.7%) in patients with a fold increase of < 1.9. For patients with a declined serum β-hCG, another serum β-hCG test should be scheduled 7–10 days later, since abnormal conception like ectopic pregnancy can occur, similar to the case of ectopic pregnancy in our present study. Shamonki et al., confirmed that declining serum β-hCG levels almost always led to a failed live birth, although they reported 3 cases of live birth with declined serum β-hCG levels in a cohort of 6021 patients [10].

A few previously reported studies investigated the prediction of pregnancy outcomes by serum β-hCG levels over various days following vitrified-warmed blastocyst transfer. Oron et al., demonstrated that for β-hCG that was measured 11 days after single blastocyst transfer, the optimal cut-off value for predicting clinical pregnancy was 137 IU/L with a PPV of 85% and an NPV of 75% [6]. The study by Xiong et al., determined that optimal thresholds were 152.2 IU/L and 211.9 IU/L respectively in predicting clinical pregnancy and live births in patients that had β-hCG tests 11 days after vitrified-warmed blastocyst transfer [7]. Zhao et al., found that the single β-hCG value of 399.5 IU/L on day 12 after blastocyst transfer was reliable to predict clinical pregnancy with a PPV of 93.47% and an NPV of 67.61%. The single β-hCG value > 410.8 IU/L indicated that 76.62% of live births and a value below that threshold, resulted in 80.72% of non-live births [8]. The thresholds predicting clinical pregnancy and live births in these studies were higher than those in our study, which can be explained by the fact than only patients with a low β-hCG were included in our research.

Stone et al., investigated the association between the doubling time of serum-borne β-hCG (β-t2) and ongoing pregnancy in pregnant women after assisted reproductive technology (ART). They illustrated that the β-t2 on day 12 after embryo transfer was about 1.6 days and the cut-off value of 2.2 days had an optimal PPV of 87% and an NPV of 42% [11]. Sung et al., calculated the fold increase between post-ovulatory day 12 and day 14 in frozen-thawed cycles, but a difference between live births and early pregnancy loss was not found (3.1 ± 0.9 folds vs. 3.0 ± 1.0 folds; *P >* 0.05). Nevertheless, they demonstrated that the 2.37- and 2.6-fold values respectively predicted 89.8% of clinical pregnancies and 72.7% of live births [1].

In our present study, the optimal threshold of a fold increase for clinical pregnancy was 1.4 with an AUC of 0.899, a sensitivity of 90.3%, and a specificity of 77.8% and a PPV of 97.4%. The value in predicting live births was 1.9, with an AUC of 0.803, a sensitivity of 88.5%, a PPV of 97.4% and an NPV of 92.3%. The fold increase in clinical pregnancies and live births in our research was lower than those found in Sung’s study. The possible reasons are as follows: First, the serum β-hCG levels in our study were measured 14 days after blastocyst transfer, which was at least two days later than that in Sung’s research. The study by Stone et al., showed that the doubling times of serum β-hCG (β-t2) increased from 1.6 days on day 12, to a doubling time 3 days on day 24 after embryo transfer [11], suggesting that β-hCG doubled more quickly in early pregnancy. Second, the study population in our research included patients with low serum levels of β-hCG, whose transferred embryos may be less potent than those from patients having normal β-hCG levels.

Our previous study investigated the likelihood of live births with serum levels of β-hCG < 100 mIU/ml 14 days after day 3 embryo transfer, which showed that the live birth rate was only 4.3%. Also in our present study, the live birth rate was 4.5% when the serum β-hCG level was < 100 mIU/ml at 14 days after blastocyst transfer (Table 2), which is comparable to that of those observations found for day 3 embryo transfer [12].

The present research possesses the following advantages. First, we only analyzed patients with low serum β-hCG levels instead of investigating all pregnant patients, which will be helpful in developing appropriate suggestions of follow-up for this group of patients. Second, all included patients had adoptively received only vitrified-warmed blastocysts and had serum β-hCG levels that were measured on exactly the same day after embryo transfer, which can increase the accuracy of serum β-hCG levels. However, the current study contained two disadvantages. First, the patients studied had a β-hCG test 14 days after embryo transfer. Moreover, the timing of the hCG test in each center was highly variable. Thus, our results may only be applicable in predicting patients that had a serum hCG test 14 days after blastocyst transfer. Prediction of patients that have had an hCG test other than 14 days should be calculated according to the hCG doubling time of 48 h, which limits its wider application. Second, the number of embryos that were successfully transferred varied from one to two. This may cause vanishing twin syndrome, which may affect initial serum β-hCG levels.

It has been reported that the rate of vanishing twin syndrome was as high as 10% after ART [13], which may affect the initial maternal serum β-hCG level. In the present research, the rate of a twin pregnancy was only 5.1% (16/312) with an initial serum β-hCG level < 300 mIU/ml. And finally, no vanishing twin syndrome occurred in the setting of a twin pregnancy, with the notable exception of 14 total miscarriages.

## Conclusions

Initially low serum β-hCG levels measured at 14 days after frozen blastocyst transfer indicated a minimal likelihood of a live birth. In addition, for patients having an initial β-hCG > 58.8 mIU/ml, then continued luteal phase support is strongly indicated. Another serum β-hCG test and ultrasound should be performed one week later. If the initial serum level of β-hCG is < 58.8 mIU/ml, then discontinued luteal phase support is suggested and assay of serum β-hCG and ultrasound can be arranged one week later.

## Data Availability

The data sets used and/or analyzed during the current study are available from the corresponding author on reasonable request.

## Abbreviations

ROC: Receiver operating characteristic
AUC: Area under the ROC curve
CI: Confidence interval
ART: Assisted Reproductive technology
HMG: Human menopausal gonadotropin
HCG: Human chorionic gonadotropin
AMH: Anti-müllerian hormone
